# User experience and hemostatic efficacy: Comparative analysis of commercial agents in junctional and hepatic hemorrhage models

**DOI:** 10.1371/journal.pone.0330696

**Published:** 2025-08-29

**Authors:** Kyle Patterson, Adelle M. Dagher, Meredith Lackie, Lauren M. Heyda, Zain Baig, John Mares, Justin Hutzler, J. T. Green, Woo Do, Jason S. Radowsky, Matthew Bradley, Brandon Propper, David M. Burmeister, Patrick Walker

**Affiliations:** 1 Battlefield Shock and Organ Support Laboratory, The Uniformed Services University of the Health Sciences, Bethesda, Maryland, United States of America; 2 Department of Surgery, The Walter Reed National Military Medical Center, Bethesda, Maryland, United States of America; 3 Department of Surgery, The Uniformed Services University of the Health Sciences, Bethesda, Maryland, United States of America; 4 Department of Medicine, The Uniformed Services University of the Health Sciences, Bethesda, Maryland, United States of America; University of Central Florida, UNITED STATES OF AMERICA

## Abstract

**Background/Objectives:**

Hemorrhage is associated with most preventable combat-related deaths. Management of non-compressible truncal and junctional hemorrhage remains challenging, especially with prolonged evacuations. This study evaluated the efficacy of commercial topical hemostatic agents in uncontrolled hemorrhage models under coagulopathic conditions, examining differences based on applicator experience.

**Methods:**

Sixty Yorkshire swine were randomized to 5 groups: Combat Gauze (CG), Celox Rapid (CR), ChitoSAM 100 (CS), EVARREST® Fibrin Sealant Patch (EP), and X-Stat 30 (XS). After 50% hemodilution, a 5 mm femoral arteriotomy or 6 cm liver laceration was created, and the agents were applied as per device instructions. Hemostatic agents were placed by experienced (≥5 previous applications) or non-experienced (<5 previous applications) users. Animals were then monitored for rebleeding for 60 minutes.

**Results:**

In the junctional hemorrhage model, only ChitoSAM required more applications by novice applicators (2.3 vs. 1, p = 0.0104). No significant differences in rebleed rates among devices (CG 0%, CS 33%, CR 17%, EP 17%, XS 33%, p = 0.73) or by user experience were observed. No differences in perfusion were noted on angiography. In the hepatic laceration model, no significant differences in applications or rebleed rates for any agent (CG 0%, CS 50%, CR 17%, EP 17%, XS 0%, p = 0.21) or by user experience were found. Overall survival did not significantly vary by device.

**Conclusion:**

All 5 hemostatic agents showed similar efficacy in controlling junctional and intra-abdominal hemorrhage, with no significant differences in hemostasis, rebleed rate, or survival by experience level. However, more ChitoSAM applications were needed for junctional hemorrhage control in the absence of experience. All tested dressings show promise for rapid hemorrhage control.

## Introduction

Rapid hemorrhage control after injury is a life-saving intervention. Most potentially preventable deaths after combat-related injury are associated with hemorrhage and occur in the prehospital phase. Non-compressible truncal hemorrhage (NCTH) is a leading cause of mortality in trauma with up to an 85% mortality rate in the military population, followed by severe extremity injury and “non-tourniquetable” junctional hemorrhage [1–3].

Non-compressible truncal and junctional hemorrhage remains a significant challenge in trauma care, especially in the setting of prolonged evacuation times during combat operations. Continued analysis of in-use hemostatic agents remains paramount. Many hemostatic agents and dressings are designed to promote rapid coagulation and arrest ongoing hemorrhage. Several characteristics of the ideal hemostatic agents include but are not limited to: quick and effective control of bleeding from a variety of wounds even when applied to an actively bleeding site, maintenance of hemostasis for several hours, simple storage and portability, biocompatibility, and easy administration by novel users under austere conditions [4–5].

The guidelines established by the Committee on Tactical Combat Casualty Care (CoTCCC) recommend the use of approved hemostatic dressings including QuikClot Combat Gauze (CG; Teleflex, Morrisville, NC), Celox Gauze (CR; Medtrade Product, United Kingdom), or ChitoGauze (Tricol Biomedical, Portland, OR) [6]. CG is the most widely used in the military setting. Several studies compared the efficacy of different hemostatic agents in various swine models of hemorrhage demonstrating their ability to effectively achieve hemostasis, reduce blood loss, and improve survival [7–11]. However, few studies focused on the individual applying the hemostatic dressing [12–13].

Experience with applying hemostatic adjuncts varies amongst personnel, highlighting the importance of ease of administration. Service members receive role-based tactical combat casualty care (TCCC) training which provides guidance for treatment from point of injury to evacuation. In pre-deployment courses medical personnel learn to treat non-compressible hemorrhage. However, in the absence of repeated exposure to actively hemorrhaging wounds, such as experienced medical personnel might encounter, these skills can atrophy. The ongoing need for hemostatic agents that are “user friendly” is paramount.

This study aims to evaluate five commercially available hemostatic agents used across a spectrum of user experience, hypothesizing that training level would have no significant effect on hemostasis. Product efficacy in terms of survival, blood loss, and durable hemostasis was assessed in swine models of junctional arterial hemorrhage and non-compressible torso hemorrhage under coagulopathic conditions.

## Methods

### Overview and study design

This study was approved by the Institutional Animal Care and Use Committee (IACUC) at the Uniformed Services University (Protocol SUR-23–112). It was conducted by the Battlefield Shock and Organ Support (BSOS) Program at USU, Bethesda, Maryland. All animal care was in strict compliance with the Guide for the Care and Use of Laboratory Animals, and the National Institutes of Health guide for the care and use of Laboratory Animals (NIH Publications No. 8023, revised 1978) in a facility accredited by the Association for the Assessment and Accreditation of Laboratory Animal Care International. All procedures were performed under appropriate anesthesia as indicated

This study used two distinct injury models: a junctional vascular arterial injury and a hepatic laceration injury. The study design for each is described with differences in protocol design noted. Healthy, adult female Yorkshire-cross swine (*Sus scrofa*), obtained from Animal Biotech Industries Inc. (Doylestown, Pennsylvania), were acclimated for a minimum of 3 days while housed at the animal vivarium to minimize environmental confounders. Female swine were used to mitigate and control for the hormonal impact and influence on the physiologic response to severe hemorrhage.

The swine were randomized into 5 groups including CG (control group) and four novel hemostatic agents (n = 30 per injury model, CG control, n = 6; per group, n = 6). The control group consisted of the current military standard, Quick Clot Combat Gauze (CG; Teleflex, Morrisville, NC). The novel hemostatic agents included Celox Rapid (CR; Medtrade Product, United Kingdom), ChitoSAM 100 (CS; Sam Medical, Tualatin, OR), EVARREST Fibrin Sealant Patch (EP; Ethicon, Raritan, NJ), and X-Stat 30 (XS; RevMedX, Wilsonville, OR). Each group was further split into experienced and non-experienced (novice) applicators for placement of the hemostatic agent. Novices had no prior training in applying topical hemostatics and had less than six months’ experience in the animal lab, while experienced applicators were all surgical providers with greater than six months’ experience. The manufacturer’s instructions for application of each agent were reviewed with both groups. The research team was not blinded to group or treatment assignment. Group size was calculated using blood loss data from a prior study (7) to achieve 90% power, with α of 0.05. As described henceforth, the animals in each injury model were subjected to the same preparation and instrumentation, baseline evaluation, hemodilution, vascular or hepatic injury, device application, and critical care phases. Arterial laboratory samples were collected during the preinjury baseline phase, at the completion of hemodilution, after injury and intervention, after a 1-hour intensive care monitoring period, and time of death. Analysis included blood gas, blood chemistry, and rotational thromboelastography (ROTEM, EXTEM)) (ABL 800 FLEX; 201 Radiometer America, Brea, CA).

### Animal preparation

All animals were female, weighing 40–75 kg. Swine were fasted for 12 hours before the study. Anesthesia was induced with 4–8 mg/kg of tiletamine/zolazepam and 2.2 mg/kg xylazine intramuscularly. The animals were intubated and maintained under anesthesia with 1–5% isoflurane and mechanically ventilated with tidal volumes of 7–10 mL/kg and a respiratory rate of 10–15 breaths per minute to maintain end-tidal carbon dioxide (CO_2_) at 40 ± 5 mmHg. Bilateral peripheral intravenous (IV) access was established in each ear, and IV fluids initiated at a rate of 2 mL/kg/hr. The swine were placed on a warming blanket to maintain temperature >36.5–39.5 °C.

### Injury and intervention

Baseline physiologic data were obtained and monitored throughout the study including blood pressure/mean arterial pressure (MAP), heart rate (HR), temperature, and oxygen saturation (O2%). The junctional vascular injury model was carried out as follows. After anesthetic induction, all swine were prepared with bilateral carotid artery cannulation, via percutaneous or open cutdown techniques, and subsequent placement of 7Fr sheaths for blood pressure monitoring using a Solid-State Pressure Catheter (Transonic Systems Inc., Ithaca, NY), hemodilution, and blood sample collection throughout the procedure. In the hepatic laceration injury model, bilateral femoral artery cannulation (as compared to carotid) via percutaneous techniques was employed. In both models, central venous access was obtained via surgical cutdown of the jugular vein and placement of a 5Fr catheter.

Following baseline laboratory evaluation, hemodilution was induced by a 50% exchange transfusion using 0.9% Sodium Chloride (NaCl) Injection (USP). Total blood volume was estimated as 65mL/kg body weight and 50% of this value was removed through the common femoral artery (liver) or carotid artery (junctional injury model) over 30 minutes, at a rate no greater than 100cc/minute. Simultaneously, an equal amount of NaCl 0.9% was injected via peripheral venous catheter.

**Fig 1:**
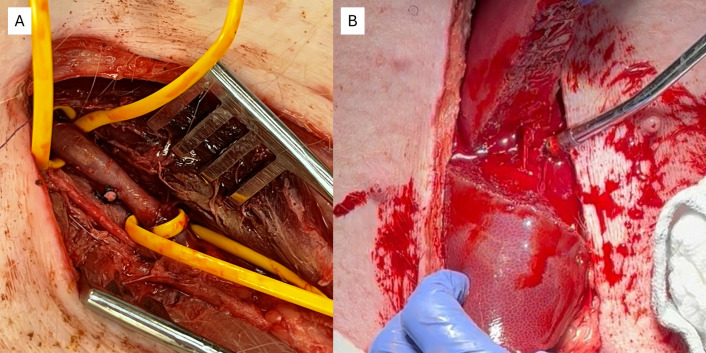
Injury Models. A) Junctional Vascular Injury, B) Hepatic Laceration.

The animals were then assigned to a hemostatic agent and an experienced or novice applicator by random number generation. Injury preparation for the penetrating junctional vascular injury model was as follows. A surgical cutdown over the right groin was performed to circumferentially dissect out the femoral artery for a length of 2–3 cm. Using vessel loops, proximal and distal control was obtained (Fig 1). A temperature probe was placed into the wound at the planned arteriotomy site. Papaverine hydrochloride (2mL) was applied to the exposed artery to promote dilation. Baseline physiologic data including MAP, HR, and temperature was obtained. An arteriotomy was made using a 5 mm aortic punch, followed by 30 seconds of free bleeding (or until mean arterial pressure (MAP) dropped below 40 mmHg) to simulate uncontrolled hemorrhage. Hemorrhage volume from the injury site was measured using a combination of suction and pre-weighted laparotomy pads.

Following the period of uncontrolled hemorrhage, hemostatic agents were applied directly to the arteriotomy with manual pressure held for a set time frame according to the manufacturer’s instructions (QuickClot Combat Gauze, ChitoSAM100, Everest Fibrin Sealant Patch, and XStat30: 3 minutes, Celox Rapid: 1-minute). Rebleeding was defined as pooling of blood around the gauze following application within a 5-minute observation period. A total of three applications of the same dressing and same applicator was allowed with a 5-minute observation following each additional application.

Injury preparation for the liver laceration was as follows. A limited upper midline laparotomy was performed, and the liver was exposed. A full thickness injury was made to the left lobe of the liver by sharply lacerating with scissors for a total length of approximately 6 cm (Fig 1). Uncontrolled hemorrhage ensued as described above. The hemostatic agent was then applied with manual pressure held (by the novice or experienced provider) to the free end of the liver for a set time frame according to the manufacturer’s instructions as above. If hemostasis (defined as the absence of any residual pooling of blood or seepage of blood around the dressing) was not achieved, or if rebleeding occurred within a 5-minute post application observation period, the dressing was removed and replaced with a new dressing of the same type with repeat compression. A total of 3 reapplications were allowed. The quantity and weight of dressings was recorded.

### Angiography and movement test

During the 1hr ICU phase, additional IV fluids and pharmacologic pressor support (phenylephrine 0.1 mg/mL) were provided to maintain MAP > 40 mmHg. The application was considered successful if the animal survived the injury, intervention, and observation period without rebleeding. In the femoral artery injury model, angiography of the injured limb was performed after the ICU period through the left carotid sheath. The limb was evaluated for extravasation, occlusion/thrombosis, and distal perfusion of the limb. Distal perfusion was documented if there was contrast flow in the extremity vasculature past the injury site. Clot stability was then assessed by performing a movement test on the injured limb by fully flexing and extending the limb at the hip and knee joint 5 times and examining for rebleeding. Clot stability in the liver laceration model was assessed by rolling the animal into left and right lateral decubitus positions 5 times and then examining the abdomen and hemostatic agent for re-bleeding. This movement was chosen to simulate pre-hospital transfer and evacuation. Prior to movement the abdominal wall edges were approximated using towel clamps to maintain abdominal contents in their position. The animal “passed” the movement test if there was no bleeding after the limb was ranged or the animal was rolled 5 times.

### Euthanasia and postmortem analysis

At the completion of the movement test, animals were humanely euthanized using pentobarbital-based euthanasia solution (Euthasol® 100 mg/kg) consistent with the 2020 American Veterinary Medical Association (AVMA) Guidelines for Euthanasia of Animals. Following euthanasia, the injured artery and tissue from the injured hind leg, or liver segment including normal uninjured liver tissue, was collected and placed in 10% Neutral Buffered Formalin. After processing through graded alcohols, paraffin embedded tissue was cut into 5µm sections and stained with hematoxylin and eosin.

### Data collection and statistical analysis

Hemodynamic and physiologic parameters were continuously measured using PowerLab data acquisition system (AD Instruments, Colorado Springs, Colorado) at a sampling rate of 1 kilo Samples per second (kS/s). The data were analyzed using LabChart V8.0 software (AD Instruments, Colorado Springs, Colorado) averages taken at 1-minute intervals across the entire recording period. The primary outcomes of the study were the number of applications required to achieve hemostasis in novice and experienced groups, survival, total blood loss, and angiography results. Secondary outcomes included incidence of re-bleeding during the movement test, and histologic changes to the injured artery, hind limb, and injured liver segment.

Continuous data are reported as means and standard error of the mean and non-continuous variables are reported as medians and interquartile range. A Two-way Analysis of Variance was conducted to examine the effects of provider experience and hemostatic agent on mortality, blood loss, and hemostasis. P < 0.05 was considered statistically significant.

## Results

A total of 60 animals were used in the experiment, with 30 animals in the junctional vascular injury group and 30 animals in the hepatic injury cohort. In each cohort, there were 6 control animals (CG) and 6 animals in each of the novel hemostatic agent groups. No animals were excluded. The baseline characteristics following hemodilution are shown in Table 1. There were no significant differences in baseline characteristics between animals. Clot formation time (CFT) and maximum clot firmness (MCF) were within accepted reference ranges [14].

**Table 1:** Baseline animal characteristics after hemodilution.

*Junctional Vascular Injury*										
	CG		CS		CR		EP		XS	
	Novice	Pro	Novice	Pro	Novice	Pro	Novice	Pro	Novice	Pro
**Weight, kg**	44.0 ± 1.0	35.3 ± 5.9	43.7 ± 5.7	37.7 ± 8.1	40.0 ± 6.6	36.3 ± 7.5	39.7 ± 6.3	34.3 ± 3.2	45.3 ± 4.5	32.0 ± 3.5
**Hematocrit, %**	24.5 ± 2.0	23.8 ± 0.6	23.4 ± 3.5	24.7 ± 0.9	23.9 ± 4.1	22.0 ± 3.7	21.6 ± 0.3	23.2 ± 3.4	22.7 ± 1.5	22.7 ± 1.4
**Clot Formation Time, s**	64 ± 4.2	62 ± 6.2	70 ± 5.3	60.5 ± 3.5	69.3 ± 4.0	76 ± 14	58 ± 8.7	68.7 ± 7	72.7 ± 14	58.3 ± 7.6
**Maximum Clot Firmness, mm**	72.5 ± 0.70	70.3 ± 5.8	66.0 ± 6.1	55.67 ± 34	69.7 ± 3.5	68.3 ± 3.8	74.0 ± 2.6	68.7 ± 2.3	69.0 ± 5	72.7 ± 1.5
***Hepatic Laceration***										
**Weight, kg**	54.3 ± 5.5	55.7 ± 6.6	58.0 ± 4.6	57.0 ± 5.0	57.3 ± 6.6	59.7 ± 11.7	52.6 ± 1.2	59.0 ± 6.9	52.7 ± 2.5	57.0 ± 6.2
**Hematocrit, %**	23.5 ± 3.2	21.3 ± 3.4	23.7 ± 5.6	21.8 ± 3.7	23.3 ± 3.3	27.4 ± 7.3	23.2 ± 3.0	21.7 ± 4.2	22.6 ± 1.5	21.9 ± 3.1
**Clot Formation Time, s**	68.5 ± 9.2	54.3 ± 24.1	62.0 ± 10.5	62.3 ± 5.5	63.3 ± 27.4	71.7 ± 14.7	57.3 ± 6.4	67.7 ± 10.1	57.3 ± 11.7	69.7 ± 9.1
**Maximum Clot Firmness, mm**	66.5 ± 0.7	73.7 ± 11.1	67.3 ± 4.2	74.7 ± 2.3	72.3 ± 6.5	67.3 ± 4.7	69.0 ± 3.6	70.7 ± 2.1	78.3 ± 5.5	67.7 ± 7.1

CG; QuickClot Combat Gauze, CS; ChitoSAM100, CR; Celox Rapid, EP; Everest Fibrin Sealant Patch, XS; X-Stat30

**Fig 2:**
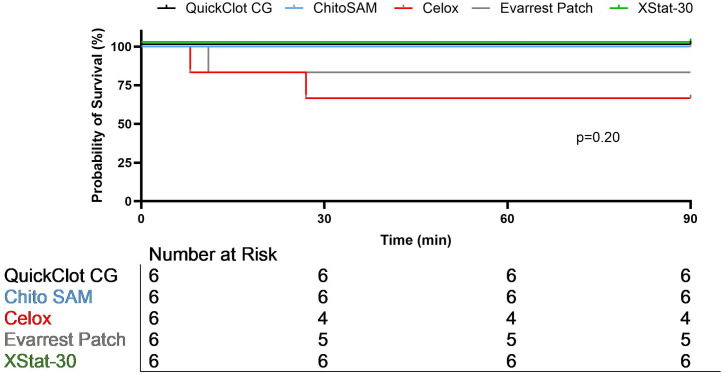
Survival in junctional vascular injury.

**Fig 3:**
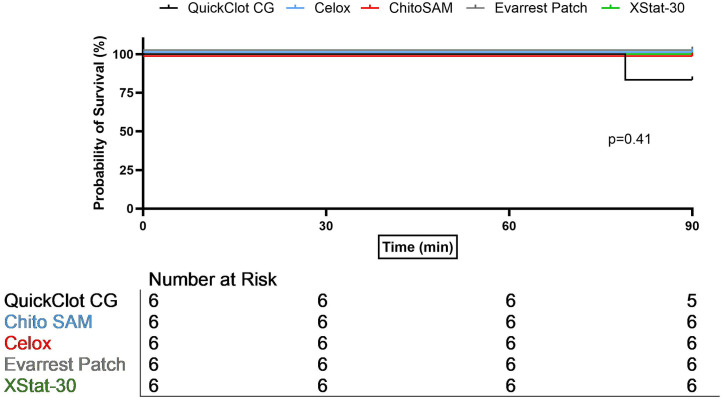
Survival in hepatic laceration.

**Fig 4:**
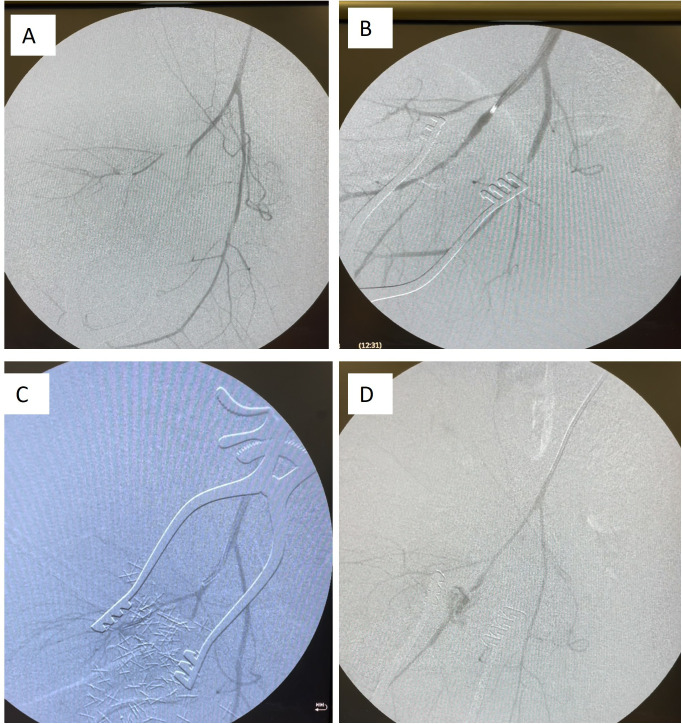
Angiograms obtained during experienced application of hemostatic agents to junctional vascular injury. **a)** Control Group (QuickClot), **b)** Everest Patch, **c)** XStat-30, **d)** ChitoSAM100.

To compare outcomes and efficacy for each novel hemostatic agent we first considered all skill levels. There was no significant difference in heart rate, lactate, hematocrit, or platelet count between treatment groups for either model, or MAP in the junctional injury model. In the hepatic injury model, the overall variance in MAP was significant (p = 0.03), but no group was significantly different than combat gauze controls (Table 2). No difference in average blood loss, rebleed rate, or overall survival among the devices was observed in either injury model (Table 3, Fig 2–3). No significant difference was found in response to the movement test in either injury model. In junctional hemorrhage, no difference in extravasation or perfusion was demonstrated on angiogram (Fig 4) and while distal occlusion occurred more frequently in the CG (100%), CS (75%) and XS (75%) groups, this difference was not significant.

**Table 2:** Hemodynamic and physiologic parameters.

*Junctional Vascular Injury*																
	CG			CS			CR			EP			XS			p
	EOH	ICU	EOE	EOH	ICU	EOE	EOH	ICU	EOE	EOH	ICU	EOE	EOH	ICU	EOE	
**HR BPM**	78 ± 17	75 ± 9.3	78 ± 16	78 ± 13	81 ± 10	110 ± 32	84 ± 12	90 ± 15	92 ± 14	74 ± 20	74 ± 30	76 ± 33	83 ± 12	90 ± 3	95 ± 25	0.33
**MAP** **mmHg**	49 ± 5.9	43 ± 7.7	54 ± 12	38 ± 8.3	38 ± 7.3	51 ± 3.4	37 ± 14	35 ± 12	51 ± 27	38 ± 8.1	37 ± 7.4	62 ± 12	43 ± 8.4	35 ± 4.7	55 ± 38	0.60
**Lactate mmol/L**	2.1 ± 0.44	2.3 ± 0.54	2.1 ± 0.56	1.7 ± 0.50	1.8 ± 0.25	2.7 ± 1.1	3.2 ± 2.0	3.4 ± 3.0	5.0 ± 4.2	0.87 ± 0.0	2.3 ± 0.0	2.90 ± 0.0	1.2 ± 0.7	1.6 ± 0.8	3.2 ± 3.7	0.87
**HCT** **%**	24 ± 1.4	24 ± 1.5	23 ± 2.8	24 ± 2.4	23 ± 1.9	23 ± 2.5	23 ± 3.7	24 ± 3.1	24 ± 3.6	22 ± 2.3	23 ± 3.2	23 ± 1.9	23 ± 1.3	22 ± 2.3	22 ± 2.9	0.49
**Platelet 10** ^**3**^ **/uL**	276 ± 94.5	277 ± 83.3	273 ± 91.2	326 ± 82.1	321 ± 51.8	311 ± 72.8	287 ± 97.7	278 ± 113	243 ± 114	294 ± 70.7	299 ± 50.1	313 ± 32.6	350 ± 128	350 ± 167	342 ± 173	0.69
***Hepatic Laceration***																
**HR BPM**	82 ± 20	85 ± 8.1	86 ± 19	88 ± 16	93 ± 21	93 ± 20	80 ± 20	93 ± 29	96 ± 27	84 ± 14	79 ± 11	92 ± 11	88 ± 19	93 ± 18	97 ± 22	0.86
**MAP** **mmHg**	55 ± 9.9	46 ± 10	48 ± 16	51 ± 4.2	45 ± 6.0	46 ± 8.0	56 ± 5.9	53 ± 7.5	56 ± 7.0	60 ± 5.6	52 ± 10	57 ± 5.8	51 ± 5.6	44 ± 9.0	55 ± 8.0	0.03
**Lactate mmol/L**	2.1 ± 0.43	2.3 ± 0.55	2.1 ± 0.56	1.7 ± 0.50	1.8 ± 0.25	2.7 ± 1.1	3.2 ± 1.96	3.4 ± 3.0	5.0 ± 4.2	0.99 ± 0.16	1.9 ± 0.57	2.1 ± 0.078	2.1 ± 1.1	2.3 ± 0.57	1.9 ± 0.36	0.51
**HCT** **%**	22 ± 3.2	24 ± 1.6	24 ± 1.4	23 ± 3.0	22 ± 2.6	23 ± 2.3	25 ± 5.5	25 ± 2.8	24 ± 2.2	22 ± 3.4	23 ± 2.6	23 ± 1.8	22 ± 2.2	25 ± 5.9	25 ± 3.3	0.59
**Platelet 10** ^**3**^ **/uL**	234 ± 109	276 ± 80.8	246 ± 89.1	272 ± 92.7	314 ± 79.2	317 ± 75.5	246 ± 68.5	239 ± 35.6	252 ± 66.2	286 ± 55.1	310 ± 82.0	305 ± 90.8	265 ± 61.5	274 ± 59.1	282 ± 61.1	0.66

CG; QuickClot Combat Gauze, CS; ChitoSAM100, CR; Celox Rapid, EP; Everest Fibrin Sealant Patch, XS; X-Stat30, EOH: End of Hemorrhage, ICU: Start of ICU, EOE: End of Experiment

**Table 3:** Outcomes and efficacy of hemostatic agents.

*Junctional Vascular Injury*						
Hemostatic Agent	CG (control)	CS	CR	EP	XS	*p*
Average blood loss (mL)	68 ± 66	171 ± 193	94 ± 99	27 ± 30	291 ± 296	0.08
Rebleed Rate (%)	0	33	20	17	33	0.73
Movement Test Pass Rate (%)	80	80	100	100	100	> 0.99
Angiographic perfusion (%)	0	25	50	60	25	0.3
***Hepatic Laceration***						
Average blood loss (mL)	71 ± 44	69 ± 42	120 ± 136	27 ± 56	51 ± 55	0.32
Rebleed Rate (%)	0	50	17	17	0	0.21
Movement Test Pass Rate (%)	100	100	100	100	100	--
CG; QuickClot Combat Gauze, CS; ChitoSAM100, CR; Celox Rapid, EP; Everest Fibrin Sealant Patch, XS; X-Stat30						

Next, we separated out experience levels in both models as shown in Table 4. CS animals required a mean of 2.3 applications to achieve hemostasis in the junctional injury model by novices compared to 1 application by experienced users (p = 0.0104). Among the other novel hemostatic agents, there was no difference in the number of gauze applications required between novice and experienced applicators. Similarly, no significant difference was found in the number of required applications for any of the agents in the hepatic laceration model.

**Table 4:** Comparison of number of required product applications to obtain hemostasis between novices and experienced users across different agents.

*Junctional Vascular Injury*					
Hemostatic Agent	CG (control)	CS	CR	EP	XS
No. gauze applications (novice)	1.7	2.3	1.7	1.3	1.3
No. gauze applications (pro)	1.0	1.0	1.0	1.7	1.7
*p*	0.17	0.01	0.17	0.49	0.49
***Hepatic Laceration***					
No. gauze applications (novice)	1.7	2.3	1.0	1.0	1.3
No. gauze applications (pro)	1.3	1.3	1.7	1.3	1.7
*p*	0.58	0.28	0.28	0.58	0.58

CG; QuickClot Combat Gauze, CS; ChitoSAM100, CR; Celox Rapid, EP; Everest Fibrin Sealant Patch, XS; X-Stat30.

**Fig 5:**
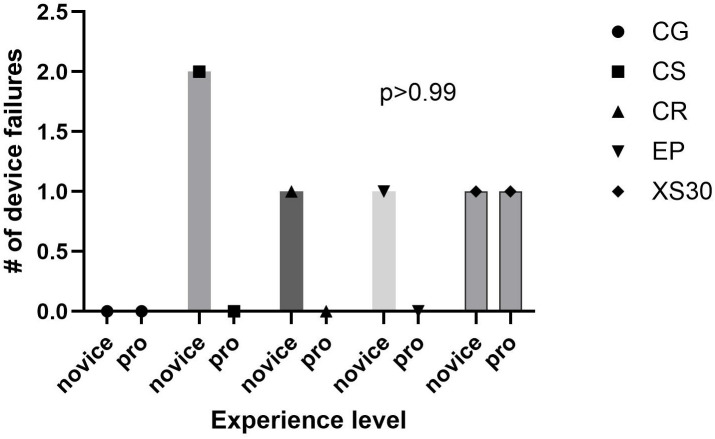
Rebleed rate by experience level- Junctional Vascular Injury. CG, QuickClot Combat Gauze; CS, ChitoSAM100, CR; Celox Rapid; EP, Everest Fibrin Sealant Patch; XS, X-Stat30.

**Fig 6:**
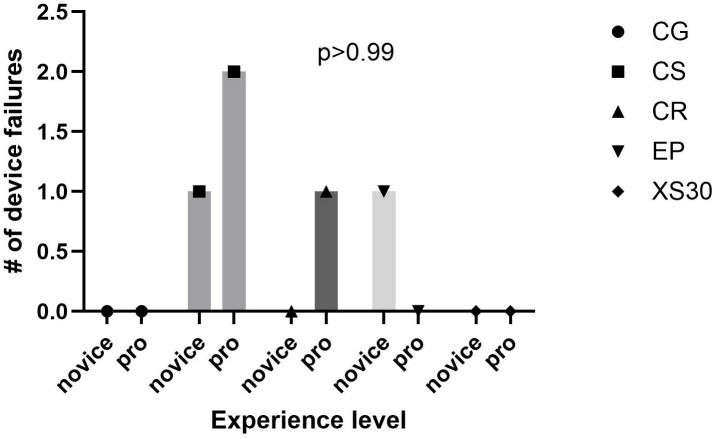
Rebleed rate by experience level- Hepatic Laceration. CG, QuickClot Combat Gauze; CS, ChitoSAM100, CR; Celox Rapid; EP, Everest Fibrin Sealant Patch; XS, X-Stat30.

Small differences in the number of rebleeding events were observed between novice and experienced applicators in both injury models as shown in Fig 5 and Fig 6. However, none of the differences in individual rebleed rates were statistically significant in either model.

## Discussion

This study aims to expand on previous work by investigating the effect of personnel experience level on the efficacy of novel hemostatic adjuncts for hemorrhage control. Previous work assessing the agents included in this study found no difference in hemostatic efficacy in a swine model of junctional hemorrhage when applied by a surgically trained responder [7]. The data presented herein demonstrate that this equipoise persists across responder experience levels, even when applied to intra-abdominal hemorrhage.

Hemorrhage control at the point of injury remains a critical aspect in survival of trauma patients [15–16]. In military pre-hospital settings, this becomes increasingly important considering the growing likelihood of prolonged transport times in large-scale combat operations. TCCC guidelines recommend QuikClot Combat Gauze, the control in this study, for its hemostatic superiority compared to standard surgical packing [10] and ease of training [6]. However, guidelines also endorse the use of Celox and ChitoGauze where required, citing comparable efficacy. Our data aligns with this recommendation, showing insignificant differences in blood loss, agent failure rate, and survival.

The critical aspect of this experiment assesses the performance of each dressing across applier experience levels. In an austere environment or mass-casualty incident, an ideal hemostatic adjunct would have excellent performance in the hands of the minimally trained, reducing resource constraints, and serving as a buffer to previously demonstrated responder fatigue while rendering aid [17]. Except for a reduced number of applications for ChitoSAM, no experimental agent, nor the combat gauze control displayed a significant reduction in performance when applied by novice users. Similarly, in both junctional and hepatic hemorrhage models, there was no significant incidence of rebleeding following a movement test replicating the demands of patient transport. These results agree with previous data showing that, in terms of clinical success, all the included hemostatic agents are effective adjuncts to hemorrhage control.

There are several limitations to consider when applying these results. Firstly, significant coagulopathy was not induced in any experimental group by hemorrhage alone. In previous models, this was achieved by the addition of hetastarch during exchange transfusion [7], which was not included in the current model. Though this omission resulted in normal ROTEM parameters, it more accurately models coagulation physiology in the moments after injury, during which these hemostatic agents are ideally applied. Secondly, this study used only female swine to avoid additional surgical manipulation required for catheterization in male animals. Similarly, pre-emptive splenectomy was not to prevent autotransfusion as recent literature shows no significant impact on coagulation parameters during hemorrhage [18], and suggests a confounding sympathetic response induced by splenectomy. Lastly, though novice and experienced users were explicitly defined, intra group variability in performance will inevitably exist in both experimental and practical settings. Time to effective hemostasis, though not assessed in this series, is an important variable for consideration in future research. Though small differences in device and personnel performance will persist, this data continues to support that these devices serve as equally effective adjuncts in hemorrhage control.

## Conclusion

The 5 hemostatic agents tested, in both the model of hepatic laceration and junctional hemorrhage, performed with similar efficacy. No difference in ability to attain hemostasis, rebleed rate, or survival was demonstrated across experience level. All tested dressings show promise for rapid hemorrhage control.

### Disclosure

The opinions and assertions expressed herein are those of the author(s) and do not necessarily reflect the official policy or position of the Uniformed Ser vices University or the Department of Defense, or The Henry M. Jackson Foundation for the Advancement of Military Medicine, Inc. Mention of trade names, commercial products, or organizations does not imply endorsement by the US Government. The authors have no financial interest related to the conduct of this research or the products tested or discussed during the project. In conducting research using animals, the investigators adhere to the laws of the United States and regulations of the Department of Agriculture
